# Non-Coding RNAs in Gastric Cancer: From Malignant Hallmarks to Clinical Applications

**DOI:** 10.3389/fcell.2021.732036

**Published:** 2021-11-03

**Authors:** Di Chen, Shuai Ping, Yushuang Xu, Mengmeng Wang, Xin Jiang, Lina Xiong, Li Zhang, Honglu Yu, Zhifan Xiong

**Affiliations:** ^1^ Department of Gastroenterology, Liyuan Hospital, Tongji Medical College, Huazhong University of Science and Technology, Wuhan, China; ^2^ Department of Orthopaedics, Liyuan Hospital, Tongji Medical College, Huazhong University of Science and Technology, Wuhan, China

**Keywords:** gastric cancer, non-coding RNAs, biological functions, biomarker, targeted therapy

## Abstract

Gastric cancer (GC) is one of the most lethal malignancies worldwide. However, the molecular mechanisms underlying gastric carcinogenesis remain largely unknown. Over the past decades, advances in RNA-sequencing techniques have greatly facilitated the identification of various non-coding RNAs (ncRNAs) in cancer cells, including microRNAs (miRNAs), long non-coding RNAs (lncRNAs), and circular RNAs (circRNAs). Accumulating evidence has revealed that ncRNAs are essential regulators in GC occurrence and development. However, ncRNAs represent an emerging field of cancer research, and their complex functionality remains to be clarified. Considering the lack of viable biomarkers and therapeutic targets in GC, further studies should focus on elucidating the intricate relationships between ncRNAs and GC, which can be translated into clinical practice. In this review, we summarize recent research progress on how ncRNAs modulate the malignant hallmarks of GC, especially in tumor immune escape, drug resistance, and stemness. We also discuss the promising applications of ncRNAs as diagnostic biomarkers and therapeutic targets in GC, aiming to validate their practical value for clinical treatment.

## Introduction

Gastric cancer (GC) is one of the most commonly diagnosed malignancies, with over one million estimated new cases annually, and remains the third most common cause of cancerrelated deaths worldwide (Bray et al., 2018). Due to occult and atypical symptoms at the early stage of GC, most patients are diagnosed at advanced stages with poor prognosis (Smyth et al., 2020). Despite improvements in treatments, including surgery, molecular targeted therapy, radiotherapy, and chemotherapy, advanced GC patients continue to have a poor outcome, and the 5-years survival rate remains pessimistic (Smyth et al., 2020; Thrift and El-Serag, 2020). Although *Helicobacter pylori* (*H. pylori*), smoking, and Epstein–Barr virus (EBV) are associated with gastric carcinogenesis (Pourhoseingholi et al., 2015; Thrift and El-Serag, 2020), the exact molecular mechanisms of GC progression are still poorly understood. Therefore, a better understanding of the molecular mechanisms of gastric oncogenesis is urgently needed and is essential for the future development of effective treatments.

**FIGURE 1:**
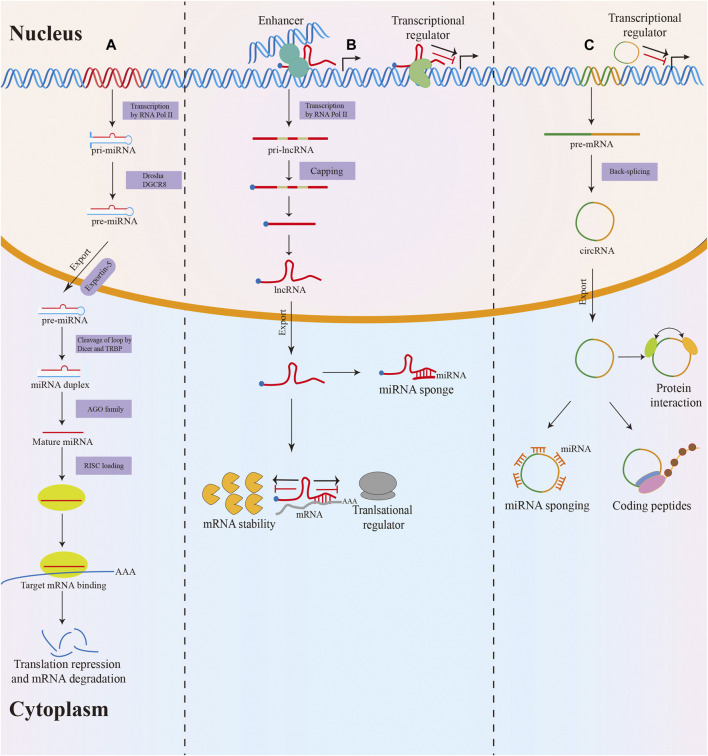
Biogenesis and function of miRNA, lncRNA and circRNA. **(A)** MiRNAs are processed from stem-loop structures of longer RNA transcripts. Mature miRNAs are loaded into the RNA-induced silencing complex (RISC) to modulate target mRNA expression by degradation or translational repression. **(B)** Most lncRNAs transcribed by RNA polymerase II are polyadenylated at 3′, 5′ capped and spliced. LncRNAs act as decoys or sponges for transcription factors or miRNAs and serve as regulatory molecules in epigenetic gene regulation, splicing, mRNA stability, and translation. **(C)** The majority of circRNAs are formed by back-splicing of precursor mRNAs (pre-mRNAs). CircRNAs exert their function via various mechanisms, including miRNA sponging, translation into proteins, interacting with proteins, and regulating their function.

Non-coding RNAs (ncRNAs) are a heterogeneous class of RNA transcripts with limited protein-coding potential that play a crucial role in the biological regulation process (Fabbri et al., 2019). NcRNAs account for more than 90% of the human genome, but most known ncRNAs have been found in the last decade and remain largely unstudied (Deveson et al., 2017; Kopp and Mendell, 2018). MicroRNAs (miRNAs), long non-coding RNAs (lncRNAs), and circular RNAs (circRNAs) are the three most studied classes of ncRNAs. MiRNAs are small ncRNAs of 19–24 nucleotides (nt) in length that are processed from stem-loop structures of longer RNA transcripts (Bartel, 2018). Functionally, most miRNAs mediate the degradation of messenger RNA (mRNA) or translational repression by binding to the 3′-untranslated region of target mRNA, thus modulating target gene expression at the posttranscriptional level (Yates et al., 2013). LncRNAs are defined as transcripts of longer than 200 nt transcribed by RNA polymerase II from independent promoters (Slack and Chinnaiyan, 2019). They have been implicated as decoys or “sponges” for transcription factors or miRNAs and acted as architectural RNAs, molecular scaffolds, or regulatory molecules in multiple cellular functions, including epigenetic gene regulation, splicing, mRNA stability, and translation (Anastasiadou et al., 2018; Yao et al., 2019). Additionally, circRNAs are a new type of endogenous ncRNAs with a covalently closed loop structure that lacks a 3′ end poly(A) tail and a 5′ end cap (Patop et al., 2019). In the past few years, multiple functions of circRNAs have been described, such as miRNA sponging, translation into proteins, interaction with proteins, and regulation of their function (Dragomir and Calin, 2018) (Figure 1).

In recent years, a growing body of evidence has revealed that ncRNAs are dysregulated in almost all types of tumors, including GC (Slack and Chinnaiyan, 2019; Sexton et al., 2020). Moreover, during the progression of GC, ncRNAs can modulate the proliferation, stemness, tumor immune escape, invasion, angiogenesis, and drug resistance of tumor cells. Therefore, the translation of ncRNA knowledge into clinical practice may bring a breakthrough. In this review, we summarize the common deregulation of ncRNAs and their roles and molecular mechanisms in regulating the malignant hallmarks of GC. Moreover, we focus on the clinical application of ncRNAs as promising biomarkers in the diagnosis, treatment, and prognosis of GC.

### Deregulation of NcRNAs in Gastric Cancer

In cancer, multiple ncRNAs are differentially expressed and show evidence of function in carcinogenesis. Among them, miRNAs have been extensively studied in various tumor types, including GC. Many well-characterized miRNAs such as miR-27a, miR-215, miR-148a, and miR-375 are significantly dysregulated in GC. MiR-27a functions as a critical oncogenic miRNA in GC, which facilitates cell proliferation and invasion via targeting prohibition or PHLPP2 (Liu et al., 2009; Ding et al., 2017). In addition, miR-27a also promotes the malignant behavior of GC cells via suppressing SFRP1 and stimulating the Wnt/β-catenin pathway (Wu et al., 2017). Oncogenic miR-215 is highly expressed in GC tissues and can be a promising biomarker for GC diagnosis (Deng et al., 2014). Functionally, miR-215 facilitates GC cell migration and invasion by inhibiting RB1 or FOXO1 expression (Chen Z. et al., 2017; Zang et al., 2017). MiR-148a, as one of the most significant tumor suppressors in GC, is related to lymph node metastasis and TNM stages (Zheng et al., 2011). Functional studies proved that miR-148a suppresses GC cell malignant behavior by repressing oncogenes, including MMP7 and ROCK1 (Zheng et al., 2011; Sakamoto et al., 2014). Tumor-suppressive miR-375 is frequently downregulated in GC and participates in the Hippo pathway via modulating YAP1/TEAD4-CTGF axis in GC cells (Kang et al., 2018). Overexpression of miR-375 suppresses the proliferation of GC cells by targeting JAK2, RON, 14-3-3zeta, and PDK1 (Ding et al., 2010; Tsukamoto et al., 2010; Lian et al., 2016).

Emerging evidence has also revealed that lncRNAs play tumor-suppressive or oncogenic roles in the progression of tumors. Aberrant expression of several lncRNAs, such as HOTAIR, H19, PVT1, and MEG3, is well-reviewed in multiple tumors, including GC. HOTAIR acts as an oncogenic lncRNA, and its expression is positively associated with TNM stages and poor survival of GC patients (Liu et al., 2014). HOTAIR facilitates GC cell proliferation and invasion via regulating the miR-1277-5p/COL5A1, miR-217/GPC5 axis, or miR-331-3p/HER2 axis (Liu et al., 2014; Dong et al., 2019; Wei Z. et al., 2020). Another study revealed that HOTAIR induces the ubiquitination of Runx3 via interacting with Mex3b, thereby resulting in enhanced invasion in GC cells (Xue et al., 2018). H19 is also an oncogenic lncRNA, which is highly expressed in GC. H19 functions as a ceRNA for miR-675 to increase RUNX1 expression, facilitating GC cell growth and proliferation (Zhuang et al., 2014). Upregulated H19 also contributes to epithelial-mesenchymal transition (EMT) and metastasis in GC by the wnt/β-catenin signaling pathway (Liu J. et al., 2021). PVT1 is significantly overexpressed in GC tissues, and its upregulation is related to poor overall survival (Xu M.-d. et al., 2017). PVT1 promotes GC cell proliferation via modulating the expression of FOXM1, p15, and p16 (Kong et al., 2015; Xu M.-d. et al., 2017). In addition, PVT1 activates the STAT3 signaling pathway to modulate the expression of VEGFA, inducing angiogenesis in GC (Zhao et al., 2018). MEG3, as a tumor-suppressive lncRNA, is remarkably downregulated in GC tissues (Ding et al., 2019). MEG3 increases Bcl-2 expression by its ceRNA activity on miR-181a, therefore suppressing GC cell proliferation, migration, and invasion. MEG3 also hinders the metastasis of GC via inhibiting the expression of miR-21 (Peng et al., 2015; Dan et al., 2018).

In recent years, differentially expressed circRNAs have been identified in multiple types of tumors, including GC. CiRS-7, one of the most studied circRNAs, has been found to play an oncogenic role in tumors. The expression of ciRS-7 is highly expressed in GC tissues and can be an independent risk factor of GC patient’s survival. Overexpression of ciRS-7 contributes to the aggressiveness of GC through blocking the miR-7-mediated PTEN/PI3K/AKT pathway (Pan et al., 2018). CircPVT1 was initially reported to be overexpressed in GC tissues. Functional studies showed that circPVT1 promotes cell proliferation via sponging members of the miR-125 family (Chen J. et al., 2017). In addition, circ-PVT1 functions as a ceRNA for miR-124-3p to upregulate ZEB1 expression, which leads to PTX resistance (Liu et al., 2019). CircMTO1 levels are much lower in GC tissues than in normal tissues. The expression of circMTO1 was correlated with tumor size, lymphatic invasion, and poor prognosis. High levels of circMTO1 enhance apoptosis and hinder invasion via acting as a miR-199a-3p sponge to increase PTEN expression (Song et al., 2020). Also, circMTO1 inhibits GC tumorigenesis by sponging miR-3200-5p to modulate PEBP1expression (Pan et al., 2018). Although there is growing interest in the role of ncRNAs in gastric carcinogenesis, more efforts are needed to explore the full extent of their contribution and the detailed functional mechanisms.

## Biological Functions of NcRNAs in Regulating the Hallmarks of Gastric Cancer

**FIGURE 2:**
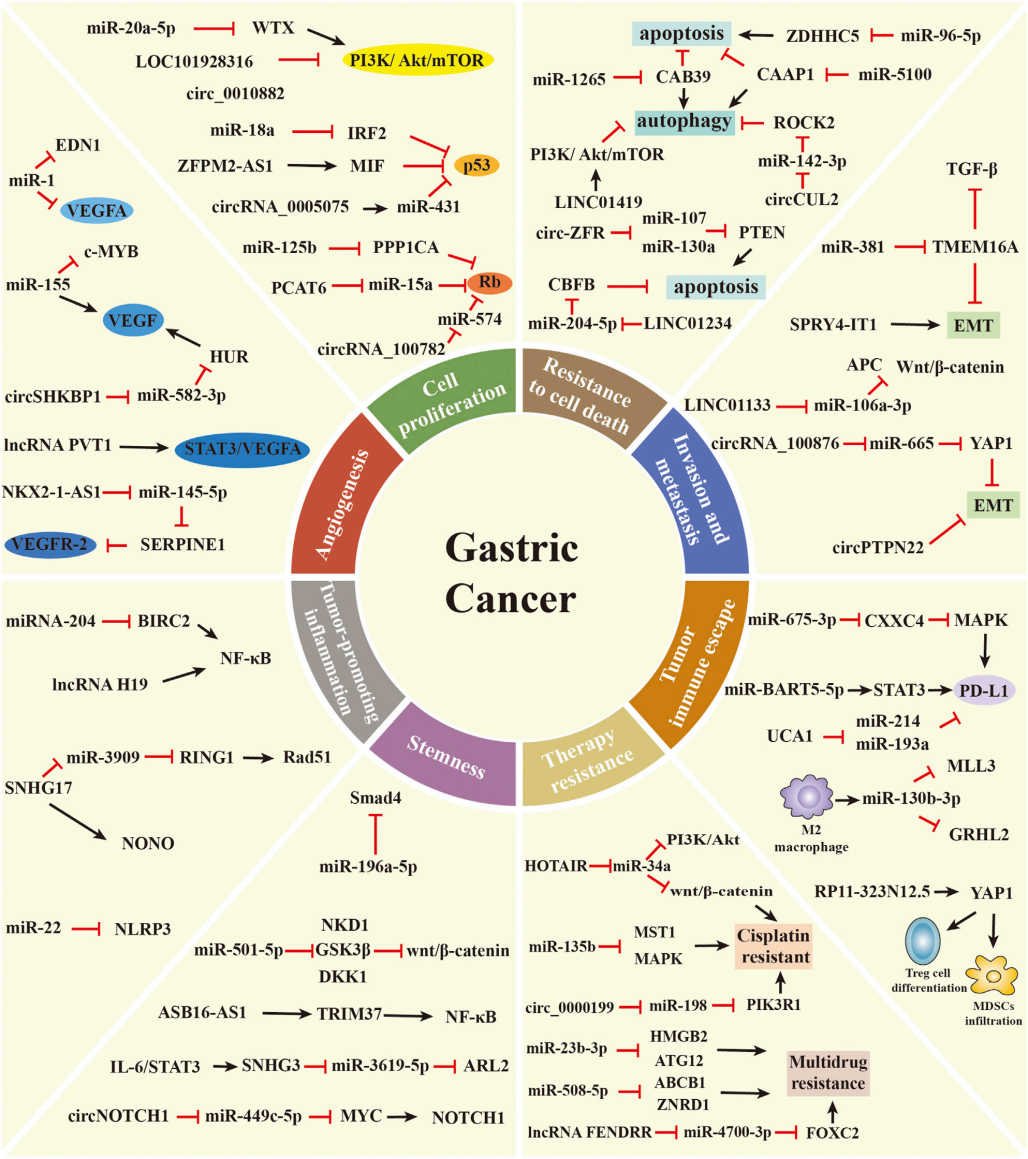
The role of ncRNAs in regulating the malignant hallmarks of GC. Selected examples of ncRNAs and their molecular mechanisms in modulating the malignant hallmarks of GC, including cell proliferation, resistance to cell death, invasion and metastasis, angiogenesis, tumor-promoting inflammation, tumor immune escape, stemness, and therapy resistance.

In 2000 and 2011, Hanahan and Weinberg proposed cancer hallmarks that lead to the progressive conversion of normal cells into tumor cells (Hanahan and Weinberg, 2000; 2011). The topic of cancer hallmarks and ncRNAs in GC has significantly grown over the past 10 years. Here, we briefly summarize the novel and well-known ncRNAs involved in modulating the malignant hallmarks of GC, especially in tumor immune escape, drug resistance, and stemness. We further present the detailed mechanisms of ncRNAs in Table 1 and Figure 2.

**TABLE 1:** The relationship between ncRNAs and the malignant hallmarks of GC.

Malignant hallmarks	RNA class	Name	Expression	Mechanisms of action	References
Proliferation	miRNA	miR-20a-5p	Up	MiR-20a-5p promotes proliferation by modulating WTX expression to affect PI3K/AKT/mTOR pathway activity	Li et al. (2020a)
		miR-18a	Up	MiR-18a regulates the expression of P53 by directly targeting IRF2, then affecting the proliferative ability	Chen et al. (2016)
		miR-125b	Up	The overexpression of miR-125b leads to Rb phosphorylation by downregulating the expression of PPP1CA.	Wu et al. (2015)
	lncRNA	LOC101928316	Down	LOC101928316 affects proliferation by regulating the PI3K/Akt/mTOR pathway	Li et al. (2019)
		ZFPM2-AS1	Up	ZFPM2-AS1 interacts with MIF to modulate the expression and subcellular localization of p53	Kong et al. (2018)
		SAMD12-AS1	Up	SAMD12-AS1 facilitates GC cell proliferation via the DNMT1/p53 axis	Lu et al. (2021)
		lncRNA PCAT6	Up	LncRNA PCAT6 changes RB/E2F and Wnt/β-catenin pathways by targeting miR-15a	Dong et al. (2020)
	circRNA	circ_0010882	Up	Circ_0010882 is capable to promote proliferation via activation of the PI3K/Akt/mTOR pathway	Peng et al. (2020b)
		circRNA_100269	Down	CircRNA_100269 suppresses the proliferation of GC cells through inactivating the PI3K/Akt pathway	Wang and Liu, (2021)
		circ_0005075	Up	Downregulation of circ_0005075 suppresses the proliferation, migration and invasion of GC cells through the miR-431/p53 axis	Wu et al. (2020)
		circ_100782	Down	Circ_100782 forms a sponge with miR-574-3p and activates tumor suppressor gene Rb	Xin and Xin, (2020)
Resistance to cell death	miRNA	miR-1265	Down	MiR-1265 modulates apoptosis by targeting CAB39 in GC and, thereby impairing oncogenic autophagy	Xu et al. (2019)
		miR-96-5p	Up	Knockdown of miR-96-5p could induce cell apoptosis by targeting ZDHHC5	Zhou et al. (2019)
		miR-5100	Down	MiR-5100 targets CAAP1 to suppress the occurrence of autophagy and promote the apoptosis of GC cells	Zhang et al. (2020a)
	lncRNA	LINC01234	Up	LINC01234 functions as a ceRNA sponge for miR-204-5p to upregulate the expression of CBFB.	Chen et al. (2018c)
		LINC01419	Up	Downregulation of LINC01419 suppresses tumor growth and promotes autophagy through inactivation of the PI3K/Akt1/mTOR pathway in GC.	Wang et al. (2019b)
		LIT3527	Up	Knockdown of lncRNA LIT3527 enhances cell apoptosis and autophagy via suppressing the AKT/ERK/mTOR signaling pathway	Wang et al. (2021a)
	circRNA	circ-ZFR	Down	Circ-ZFR promotes GC cell apoptosis through sponging miR-107/miR-130a and regulating PTEN expression	Liu et al. (2018b)
		circCUL2	Down	CircCUL2 regulates cisplatin resistance through miR-142-3p/ROCK2-mediated autophagy activation	Peng et al. (2020a)
		circ_0021087	Down	Highly expressed circ_0021087 induces GC cell apoptosis via the miR-184/FOSB axis	Yu et al. (2021)
Invasion and metastasis	miRNA	miR-381	Down	MiR-381 suppresses TGF-β signaling pathway and downregulated EMT phenotype partially by mediating TMEM16A	Cao et al. (2017)
	lncRNA	LINC01133	Down	LINC01133 suppresses the EMT and metastasis of GC by acting as a ceRNA for miR-106a-3p to modulate APC expression and the Wnt/β-catenin pathway	Yang et al. (2018)
		SPRY4-IT1	Down	SPRY4-IT1 contributes to gastric cancer cells metastasis partly through modulating EMT process	Xie et al. (2015)
		LINC01235	Up	Upregulated LINC01235 promotes the metastasis of GC cells via inducing EMT.	Zhang et al. (2021)
	circRNA	circ_100876	Up	The overexpression of circ_100876 induces the downregulation of miR-665 which in turn leads to the derepression of YAP1	Lin et al. (2020)
		circPTPN22	Up	CircPTPN22 can regulate the proliferation and metastasis of GC cells by affecting the EMT pathway	Ma et al. (2021)
Angiogenesis	miRNA	miR-1	Down	MiR-1 is capable to inhibit angiogenesis by directly suppressing VEGFA and EDN1 expression	Xie et al. (2018)
		miR-155	Up	MiR-155 obviously suppresses c-MYB but increased VEGF expression, which promoted growth, metastasis, and tube formation of vascular cells	Deng et al. (2020)
	lncRNA	lncRNA PVT1	Up	High level of PVT1 can bind to nuclear phospho-STAT3 to form a complex that increased its protein stability in the polyubiquitin-proteasome, and then activates the STAT3/VEGFA axis to stimulate angiogenesis	Zhao et al. (2018)
		NKX2-1-AS1	Up	NKX2-1-AS1 directly targets miR-145-5p to upregulate SERPINE1, leading to increased activation of the VEGFR-2 signaling pathway, thereby promoting tumor progression and angiogenesis in GC.	Teng et al. (2021)
	circRNA	circSHKBP1	Up	CircSHKBP1 sponges miR-582-3p to increase HUR expression, enhancing VEGF mRNA stability	Xie et al. (2020a)
Tumor-promoting inflammation	miRNA	miRNA-204	Down	Downregulation of miRNA-204 induced by H.pylori infection enhances expression of the target gene BIRC2, which promoted GC development and progression	Chen et al. (2020)
		miR-22	Down	MiR-22 directly targetes NLRP3, which suppresses gastric epithelial cells proliferation, attenuated inflammation and maintained homeostasis in gastric mucosa	Li et al. (2018)
	lncRNA	SNHG17	Up	Spatially independent deregulation of the SNHG17/miR-3909/RING1/Rad51 and SNHG17/NONO pathways upon *H. pylori* infection promotes the occurrence of GC.	Han et al. (2020)
		lncRNA H19	Up	High level of lncRNA H19 induced by *H. pylori* infection facilitates GC cell proliferation and invasion via enhancing NF-κB-induced inflammation response	Zhang et al. (2019)
Tumor immune escape	miRNA	miRNA-675-3p	Up	EV-mediated miR-675-3p directly targets CXXC4 and upregulates PD-L1 via the MAPK signaling pathway, thus inducing the inhibitory effect of GC cells on T cell activation	Li et al. (2020b)
		miR-BART5-5p	Up	MiR-BART5-5p suppresses the target gene PIAS3 expression and increase the expression of PD-L1 through promoting the phosphorylation of STAT3	Yoon et al. (2020)
		miR-105-5p	Down	Overexpression of miR-105-5p decreases the expression of PD-L1, and induces the activation of CD8 + T cell, therefore controlling the immunogenicity of GC cells	Miliotis and Slack, (2021)
		miR-1290	Up	EV-mediated miR-1290 suppresses the proliferation of T cells via the Grhl2/ZEB1/PD-L1 pathway, resulting in immune escape of GC.	Liang et al. (2021)
		miR-16-5p	Down	Exosomal miR-16-5p derived from M1 macrophages facilitates T cells activation and inhibits GC proliferation via blocking PD1/PDL1 checkpoints	Munson et al. (2019)
		miR-107	Up	GC-derived exosomes carrying miR-107 are able to promote MDSCs expansion and activation via targeting DICER1 and PTEN, therefore promoting tumor growth and augmenting immunotherapy efficacy	Ren et al. (2019)
		miR-130b	Up	MiR130b contributes to the progression of GC by stimulating gastric epithelial cell proliferation and creating a permissive immune microenvironment	Ding et al. (2020)
		miR-130b-3p	Up	EVs loaded with miR-130b-3p mediate communication between GC cells and M2 macrophages in the tumor microenvironment through modulating MLL3 and GRHL2	Zhang et al. (2020c)
	lncRNA	UCA1	Up	UCA1 acts as a “miRNA sponge” to absorb miR-214 and miR-193a and protect the expression of PD-L1 at RNA level	Wang et al. (2019a)
		SNHG15	Up	LncRNA SNHG15 upregulates the expression of PD-L1 via sponging of miR-149-5p, therefore regulating the immune escape of GC.	Dang et al. (2020)
		lncRNA RP11-323N12.5	Up	RP11-323N12.5 enhances YAP1 transcription in GC cells and further contributes to immunosuppression through promoting MDSCs infiltration and Treg cell differentiation	Wang et al. (2021b)
		linc-POU3F3	Up	Linc-POU3F3 promotes the distribution of Tregs via activating TGF-beta signal pathway, leading to the proliferation of GC cells	Xiong et al. (2015)
The stemness of gastric cancer	miRNA	miR-196a-5p	Up	MiR-196a-5p is highly expressed in CD44 + cells and its inhibition could reduce colony formation and invasion of gastric cancer stem cells via targeting Smad4	Pan et al. (2017)
		miR-98	Down	Elevated miR-98 inhibits the ability of self-renewal, tumorigenicity, and invasion of GCSCs by targeting BCAT1	Zhan et al. (2021)
		miR-375	Down	MiR-375 acts as a tumor suppressive miRNA that reduces GC cells stemness primarily via triggering SLC7A11-dependent ferroptosis	Ni et al. (2021)
		miR-501-5p	Up	MiR-501-5p induces activation of wnt/β-catenin signaling pathway through directly targeting NKD1, GSK3β, and DKK1, which enhances the stemness phenotype in GC.	Fan et al. (2016)
		miR-98	Up	Elevated miR-98 inhibits the ability of self-renewal, tumorigenicity, and invasion of GCSCs by targeting BCAT1	Zhan et al. (2021)
		miR-375	Down	MiR-375 acts as a tumor suppressive miRNA that reduces GC cells stemness primarily via triggering SLC7A11-dependent ferroptosis	Ni et al. (2021)
	lncRNA	FEZF1-AS1	Up	Knockdown of FEZF1-AS1 attenuates the proliferation, migration, and viability of GCSCs via the miR-363-3p/HMGA2 axis	Hui et al. (2020)
		lncRNA ASB16-AS1	Up	ASB16-AS1 activates NF-κB pathway via inducing TRIM37 phosphorylation to facilitate tumor growth, stemness, and cisplatin resistance in GC.	Fu et al. (2021)
		LncRNA ADAMTS9-AS2	Down	LncRNA ADAMTS9-AS2, as a tumor suppressor, inhibits the tumorigenicity of GCSCs through modulating SPOP expression	Wang et al. (2020a)
		THOR	Up	THOR contributes to the stemness of GC cells through increasing stemness marker SOX9 mRNA stability	Song et al. (2018)
		lncRNA SNHG3	Up	SNHG3, induced by IL-6/STAT3 transactivation, predominantly plays its oncogenic properties in GC by modulating the miR-3619-5p/ARL2 axis	Sun et al. (2021a)
	circRNA	circFAM73A	Up	High levels of circFAM73A increases CSC-like properties via regulating miR-490-3p/HMGA2 pathway, therefore leading to GC malignancy	Xia et al. (2021)
		circLMP2A	Up	CircLMP2A plays an essential role in inducing and maintaining stem cell-like phenotype by targeting the miR-3908/TRIM59/p53 axis in EBV-associated GC cells	Gong et al. (2020)
		circNOTCH1	Up	CircNOTCH1 promotes metastasis and stemness via the miR-449c-5p/MYC/NOTCH1 axis	Zhao et al. (2020)
Drug resistance	miRNA	miR-21	Up	MiR-21 induces CDDP resistance via inhibiting the expression of PTEN and activation of Akt pathway	Yang et al. (2013)
		miR-99a and miR-491	Up	Oncogenic miR-99a and miR-491 enhance CDDP resistance of GC cells by directly suppressing their target gene CAPNS1	Zhang et al. (2016)
		miR-223	Up	The overexpression of miR-223 promotes CDDP resistance of GC cells by modulating cell cycle via targeting FBXW7	Zhou et al. (2015)
		miR-7	Down	MiR-7, as a tumor suppressor, also increases CDDP sensitivity via targeting mTOR in GC cells	Xu et al. (2017b)
		miR-129	Down	Knockdown of miR-129 reduces chemosensitivity to CDDP via supressing the expression of P-gp in GC cells	Lu et al. (2017)
		miR-23b-3p	Down	The overexpression of miR-23b-3p suppresses autophagy in MDR cells by targeting HMGB2 and ATG12 and enhances chemotherapy sensitivity of MDR cells	An et al. (2015)
		miR-495-3p	Down	High levels of miR-495-3p inhibit the MDR of GC cells by targeting GRP78 and modulating autophagy process	Chen et al. (2018b)
		miR-508-5p	Down	MiR-508-5p is identified as a MDR-related miRNA, which reverses GC cell resistance to multiple chemotherapeutics by suppressing the expression of ABCB1 and ZNRD1	Shang et al. (2014)
		miR-19a/b	Up	MiR-19a/b regulates MDR of GC cells via the PTEN/AKT signaling pathway	Wang et al. (2013)
		miR-106a	Up	Overexpression of miR-106a induces MDR via downregulation of RUNX3	Zhang et al. (2013)
		miR-1	Down	MiR-1 reverses MDR in GC by inhibiting sorcin expression	Deng et al. (2019)
	lncRNA	lncRNA HOTAIR	Up	LncRNA HOTAIR contributes to CDDP resistance of GC cells via sponging endogenous miR-34a to inhibit PI3K/Akt and Wnt/β-catenin signaling pathways	Cheng et al. (2018)
		HOXD-AS1	Up	HOXD-AS1 leads to CDDP resistance in GC through epigenetically silencing PDCD4 expression	Ye et al. (2019)
		FOXD1-AS1	Up	FOXD1-AS1 promotes the translation of FOXD1 via PIK3CA/PI3K/AKT/mTOR signaling, therefore inducing GC chemotherapy resistance	Wu et al. (2021)
		lncRNA CRAL	Down	lncRNA CRAL acts as a competing endogenous RNA to reverse CDDP resistance in GC via the miR-505/CYLD/AKT axis	Wang et al. (2020b)
		ADAMTS9-AS2	Down	ADAMTS9-AS2 functions as a tumor suppressive lncRNA and sensitizes GC cells to CDDP through inducing NLRP3 mediated pyroptotic cell death via downregulating miR-223-3p	Ren et al. (2020)
		MRUL	Up	MRUL plays an enhancer-like role in upregulating P-gp expression, leading to the development of MDR.	Wang et al. (2014)
		lncRNA FENDRR	Up	Downregulation of FENDRR decreases drug resistant of MDR GC cells by performing an enhancer-like role and sponging miR-4700-3p to promote FOXC2 expression	Liu et al. (2021a)
	circRNA	circMCTP2	Down	High levels of circMCTP2 sensitize GC cells to CDDP through miR-99a-5p-mediated induction of MTMR3 expression	Sun et al. (2020)
		circAKT3	Up	Knockdown of circAKT3 effectively increases CDDP sensitivity in GC cells via targeting the miR-198/PIK3R1 axis	Huang et al. (2019)
		circCUL2	Down	CircCUL2 functions as a regulator of cisplatin sensitivity via miR-142-3p/ROCK2-mediated autophagy activation	Peng et al. (2020a)
		circDONSON	Up	Silencing of circDONSON reduces CDDP resistance of GC cells through modulating the miR-802/BMI1 axis	Liu et al. (2020)

### Cell Proliferation

Uncontrolled cell proliferation is one of the most critical cancer hallmarks. Tumor cells can sustain uncontrolled proliferative states by inducing proliferative signaling pathways, such as the phosphatidylinositol 3-kinase (PI3K) pathway (Hanahan and Weinberg, 2011; Hanker et al., 2019). The PI3K pathway is evolutionarily conserved from yeast to mammals. In higher eukaryotes, the PI3K pathway plays a crucial role in modulating diverse cellular processes, including proliferation, survival, metabolism, and growth. The pathologic activation of this pathway is one of the most common events in human cancers. Various ncRNAs have been found to regulate PI3K pathways affecting GC cell proliferation. Li et al. revealed that miR-20a-5p promotes GC cell proliferation by modulating WTX expression to affect PI3K/AKT/mTOR pathway activity (Li J. et al., 2020). LncRNA-LOC101928316 is downregulated in GC samples, and its low levels are associated with the degree of differentiation and TNM stage. Functional studies proved that LOC101928316 affects proliferation by regulating the PI3K/Akt/mTOR pathway (Li et al., 2019). CircRNA_100269 is also remarkably downregulated in GC samples, and its expression is an independent risk factor to predict GC patient’s survival. Mechanistically, circRNA_100269 suppresses the proliferation of GC cells through inactivating the PI3K/Akt pathway (Wang and Liu, 2021). Moreover, Peng et al. discovered that circ_0010882 is overexpressed in the plasma of GC patients and GC cell lines. *In vitro* and *in vivo* experiments revealed that this circRNA is capable of promoting proliferation via activating the PI3K/Akt/mTOR pathway (Peng YK. et al., 2020).

In addition to their capability of inducing and sustaining proliferative signals, cancer cells also avoid the growth-suppressive effects of tumor suppressors (Hanahan and Weinberg, 2011). For example, p53 is one of the most common tumor suppressors in human cancers. The function of p53 has been attributed to its ability to permanently inhibit cell proliferation or promote cell death (Kruiswijk et al., 2015). Chen et al. observed that miR-18a is upregulated in GC tissues. Overexpressing this miRNA in GC cell lines promotes cell proliferation, migration, and invasion. Mechanistically, miR-18a regulates the expression of P53 by directly targeting IRF2, thereby affecting the proliferative ability of GC cells (Chen et al., 2016). LncRNA ZFPM2-AS1 expression is also significantly upregulated in GC and positively associated with poor survival of GC patients. The knockdown of ZFPM2-AS1 inhibits the proliferation and blocks the cell cycle of GC cells. ZFPM2-AS1 interacts with MIF to modulate the expression and subcellular localization of p53 (Kong et al., 2018). Furthermore, the downregulation of circRNA_0005075 suppresses the proliferation, migration, and invasion of GC cells through the miR-431/p53 axis (Wu et al., 2020). The critical tumor suppressor Rb is well known for its ability to prevent cell proliferation via blocking cells either in the G1, G1/S transition, or S phase of the cell cycle (Manning and Dyson, 2012). In GC, upregulated miR-125b leads to Rb phosphorylation by downregulating the expression of PPP1CA, thus promoting cell proliferation, migration, and invasion *in vitro* (Wu et al., 2015). SAMD12-AS1 is highly expressed in GC tissues and cell lines. *In vitro* experiments showed that SAMD12-AS1 facilitates GC cell proliferation via the DNMT1/p53 axis (Lu et al., 2021). Also, lncRNA PCAT6 is remarkably overexpressed in GC tissues compared with adjacent non-cancer tissues. The silencing of lncRNA PCAT6 changes RB/E2F and Wnt/β-catenin pathways by targeting miR-15a, inhibiting the proliferation and EMT of GC cells (Dong et al., 2020). CircRNA_100782 is downregulated in GC cells and is associated with cell proliferation and invasion. The underlying mechanism involves circRNA_100782 forming a sponge with miR-574-3p and activating the tumor suppressor gene Rb (Xin and Xin, 2020).

### Resistance to Cell Death

Cell death is a crucial biological process for physiological growth and development. The deregulation of cell death is involved in diverse diseases, including cancer (Degterev and Yuan, 2008). Apoptosis, autophagy, and necrosis are three classical forms of cell death (Chen Q. et al., 2018). The cascade of events due to ncRNAs involved in tumorigenesis is closely associated with apoptosis and autophagy. Apoptosis is activated in case of intrinsic or extrinsic stressors, which plays an antitumorigenic role (Verbrugge et al., 2010). Autophagy can sustain metabolism and homeostasis by capturing and degrading intracellular components, which is an oncogenic and antitumorigenic process (White, 2015; Amaravadi et al., 2019). Xu et al. discovered that miR-1265 expression is lower in GC tissue than in the adjacent normal mucosa. Further experiments revealed that miR-1265 modulates apoptosis by targeting CAB39 in GC, thereby impairing oncogenic autophagy (Xu et al., 2019). Zhou et al. found that the levels of miR-96-5p are significantly increased in GC tissue, and the knockdown of miR-96-5p induces cell apoptosis by targeting ZDHHC5 (Zhou et al., 2019). Furthermore, miR-5100 can target CAAP1 to suppress the occurrence of autophagy and promote the apoptosis of GC cells (Zhang H.-M. et al., 2020). LINC01234, a novel GC-associated lncRNA, is significantly overexpressed in GC tissues. *In vitro* experiment showed that the knockdown of LINC01234 leads to growth arrest and apoptosis via the miR-204-5p/CBFBaxis (Chen X. et al., 2018). LncRNA LIT3527 levels are much higher in GC tissues than in normal tissues. Knockdown of lncRNA LIT3527 enhances cell apoptosis and autophagy via suppressing the AKT/ERK/mTOR signaling pathway (Wang B. et al., 2021). LINC01419 is also found to be highly expressed in GC tissues and cells. The downregulation of LINC01419 suppresses tumor growth and promotes autophagy through the inactivation of the PI3K/Akt1/mTOR pathway in GC (Wang L.-L. et al., 2019). Additionally, Liu et al. observed that circ-ZFR promotes GC cell apoptosis through sponging miR-107/miR-130a and regulating PTEN expression (Liu T. et al., 2018). Peng et al. demonstrated that circCUL2 modulates GC malignant transformation through miR-142-3p/ROCK2-mediated autophagy activation (Peng L. et al., 2020). Recently, Yu et al. revealed that highly expressed circ_0021087 induces GC cell apoptosis via the miR-184/FOSB axis (Yu et al., 2021).

### Invasion and Metastasis

Cancer cells undergo a multistep process to develop the capacity of invasion and metastasis (Hanahan and Weinberg, 2011). This process, which is crucial in organogenesis and metastasis, is called EMT (Mani et al., 2008; De Craene and Berx, 2013). Increasing evidence has revealed that ncRNAs can promote tumor progression by activating the invasion–metastasis cascade, wherein the EMT plays an important role. MiR-381 inhibits the migration and invasion of GC cells *in vitro* and *in vivo*. Mechanistically, miR-381 suppresses the TGF-β signaling pathway and EMT phenotype partially by mediating TMEM16A (Cao et al., 2017). LINC01133 is downregulated in GC tissues and cell lines, and its expression is associated with the progression and metastasis of GC. *In vivo* and *in vitro* experiments proved that LINC01133 functions as an anti-proliferative and anti-metastatic lncRNA. LINC01133 suppresses the EMT and metastasis of GC by acting as a ceRNA for miR-106a-3p to modulate APC expression and the Wnt/β-catenin pathway (Yang et al., 2018). Similarly, SPRY4-IT1 expression is decreased in GC tissues and correlated with the depth of invasion, advanced pathological stage, and lymphatic metastasis. Knockdown of SPRY4-IT1 contributes to GC cell metastasis partly through modulating the EMT process (Xie et al., 2015). In contrast, upregulated LINC01235 promotes the metastasis of GC cells via inducing EMT (Zhang et al., 2021). CircRNAs are also involved in the metastasis of GC. Lin et al. discovered that circRNA_100876 promotes GC cell proliferation, invasion, and migration by suppressing the EMT pathway. Mechanistically, the overexpression of circRNA_100876 induces the downregulation of miR-665, leading to the derepression of YAP1 (Lin et al., 2020). In addition, Ma et al. demonstrated that circPTPN22 regulates the proliferation and metastasis of GC cells by affecting the EMT pathway (Ma et al., 2021).

### Angiogenesis

Angiogenesis, the formation of new blood vessels from preexisting blood vessels via sprouting, is essential for tumor growth and progression (Ramjiawan et al., 2017). The vascular endothelial growth factor (VEGF) family of growth factors and their receptors play intricate roles in initiating and promoting tumor angiogenesis. VEGFA is one of the most potent inducers of angiogenesis, which can induce the proliferation and migration of endothelial cells to form new blood vessels (Yu and Wang, 2018). VEGFR2, a primary VEGFA receptor, is the crucial molecule of VEGF signaling in tumor angiogenesis (Dvorak, 2002). Recently, diverse ncRNAs have been shown to influence angiogenesis by regulating the expression of VEGF or other angiogenic factors. Xie et al. discovered that miR-1 is downregulated in GC tissues and cell lines. *In vitro* studies proved that this miRNA inhibits angiogenesis by directly suppressing VEGFA and EDN1 expression (Xie et al., 2018). Deng et al. proved the promotional effect of exosome-delivered miR-155 on tumor growth and angiogenesis in GC by using a co-culture of SGC exosomes and human umbilical vein endothelial cells. Mechanistically, miR-155 suppresses c-MYB but increases VEGF expression, which promotes the growth, metastasis, and tube formation of vascular cells (Deng et al., 2020). LncRNA PVT1 is upregulated and remarkably correlated with high microvessel density in GC. High levels of PVT1 can bind to nuclear phospho-STAT3 to form a complex that increases its protein stability and then activates the STAT3/VEGFA axis to stimulate angiogenesis (Zhao et al., 2018). Furthermore, NKX2-1-AS1 directly targets miR-145-5p to upregulate SERPINE1, leading to the activation of the VEGFR-2 signaling pathway, thereby promoting tumor progression and angiogenesis in GC (Teng et al., 2021). Xie et al. revealed that circSHKBP1 is upregulated in GC patients and correlated with vascular invasion and poor prognosis. *In vitro* and *in vivo* experiments demonstrated that the overexpression of circSHKBP1 facilitates GC cell proliferation, invasion, and angiogenesis. Mechanistically, circSHKBP1 sponges miR-582-3p to increase HUR expression, enhancing VEGF mRNA stability (Xie M. et al., 2020).

### Tumor-Promoting Inflammation

Tumor-promoting inflammation is one of the enabling features in the progression of cancer (Crusz and Balkwill, 2015). Chronic, persistent, dysregulated, and unresolved inflammation increases the risk of multiple cancers (Crusz and Balkwill, 2015). GC is frequently linked to chronic inflammation, as *H. pylori* infection is the primary causal factor in 90% of GC cases (Piazuelo et al., 2019). MiRNAs play a crucial role in the control of inflammatory response correlated with *H. pylori* infection. Matsushima et al. compared the expression levels of miRNAs in *H. pylori*-negative and *H. pylori*-positive samples and identified 55 differentially expressed miRNAs, including miR-204, miR-200a/b/c, and miR-223 (Matsushima et al., 2011). Among them, miR-204 is downregulated in *H. pylori*-infected gastric mucosal cells and shows gradually reduced expression from superficial gastritis to intestinal metaplasia. The downregulation of miR-204 induced by *H. pylori* infection enhances the expression of the target gene BIRC2. BIRC2 overexpression activates NF-κB and its downstream signaling pathways, including inflammatory processes, which promote GC development and progression (Chen et al., 2020). Furthermore, Li et al. found that miR-22 is downregulated in gastric tissue of patients with *H. pylori* infection and GC. Functionally, miR-22 directly targets NLRP3, which suppresses gastric epithelial cell proliferation, attenuates inflammation, and maintains homeostasis in the gastric mucosa (Li et al., 2018). Recently, several studies have screened and clarified the functions of lncRNAs that are differentially expressed in *H. pylori*-related GC. For example, SNHG17 is significantly upregulated in *H. pylori*-positive atrophic gastritis and GC. SNHG17 can regulate the genomic stability of GC cells, thus affecting the progression of *H. pylori*-induced GC. Mechanistic evidence revealed that spatially independent deregulation of the SNHG17/miR-3909/RING1/Rad51 and SNHG17/NONO pathways upon *H. pylori* infection promotes the occurrence of GC (Han et al., 2020). LncRNA H19 is well expressed in *H. pylori*-infected GC cells and tissues. High levels of lncRNA H19 induced by *H. pylori* infection facilitate GC cell proliferation and invasion via enhancing NF-κB-induced inflammation response (Zhang et al., 2019). Taken together, the dysregulation of ncRNAs by *H. pylori* infection may interpret at least one of the missing links between inflammation and cancer.

### Tumor Immune Escape

Tumor immune escape refers to the phenomenon by which tumor cells escape from the recognition and attack of the immune system via various mechanisms, leading to tumor growth and metastasis (Vinay et al., 2015; Jiang et al., 2019). Tumor-induced immunosuppression has become the most extensively studied mechanism of tumor immune escape. Tumor-induced immunosuppression occurs by inducing the expression of immunosuppressive molecules or their receptors, including programmed death-ligand 1/programmed death-1 (PD-L1/PD-1), which inactivate T lymphocytes to achieve tumor immune escape (Jiang et al., 2019). Miliotis et al. identified miR-105-5p as a mediator of immune modulation in GC. The overexpression of miR-105-5p decreases the expression of PD-L1 and induces the activation of CD8^+^ T cells, therefore controlling the immunogenicity of GC cells (Miliotis and Slack, 2021). Li et al. revealed that miR-675-3p delivered by GC-secreted extracellular vesicles (EVs) is involved in the immune escape of GC. EV-mediated miR-675-3p directly targets CXXC4 and upregulates PD-L1 via the MAPK signaling pathway, thus inducing the inhibitory effect of GC cells on T cell activation (Li P. et al., 2020). In addition, Liang et al. discovered that miR-1290 expression is enriched in EVs derived from GC cells. EV-mediated miR-1290 suppresses the proliferation of T cells via the Grhl2/ZEB1/PD-L1 pathway, resulting in the immune escape of GC (Liang et al., 2021). Furthermore, exosomal miR-16-5p derived from M1 macrophages facilitates T cell activation and inhibits GC proliferation via blocking PD1/PDL1 checkpoints (Munson et al., 2019). In EBV-associated GC, miR-BART5-5p is significantly downregulated and directly associated with worse clinical outcomes in patients. Additionally, miR-BART5-5p suppresses the expression of the target gene PIAS3 and upregulates the expression of PD-L1 by promoting STAT3 phosphorylation. This contributes to the proliferation, antiapoptosis, migration, and immune escape of GC cells (Yoon et al., 2020). The function of lncRNAs in GC immune escape is not completely understood, but mechanistic studies begin to emerge. As an onco-lncRNA, UCA1 promotes GC cell proliferation, migration, and immune escape. Mechanistically, UCA1 acts as a ceRNA sponge to absorb miR-214 and miR-193a and protects the expression of PD-L1 at the RNA level (Wang C.-J. et al., 2019). LncRNA SNHG15 is highly expressed in GC tissues and associated with tumor immune escape. Mechanistic evidence showed that SNHG15 upregulates the expression of PD-L1 via sponging of miR-149-5p (Dang et al., 2020).

Tumor-induced immunosuppression also involves promoting immunosuppressive cells to accumulate around the tumor and secrete immunosuppressive factors, which reduce the immune tolerance of tumor cells by suppressing cytolytic T lymphocytes, including M2 macrophages, myeloid-derived suppressor cells (MDSCs), and regulatory T cells (Treg cells) (Gabrilovich, 2017; Jiang et al., 2019). Ding et al. discovered that miR130b produced by SLFN4^+^-MDSCs plays a crucial role in the function of MDSCs, and its levels in the blood are associated with metaplastic changes in the stomach. Moreover, miR130b is well expressed in GC cells and surrounding immune cells compared with normal tissue. MiR130b might contribute to the progression of GC by stimulating gastric epithelial cell proliferation and creating a permissive immune microenvironment (Ding et al., 2020). GC-derived exosomes carrying miR-107 are able to promote MDSC expansion and activation via targeting DICER1 and PTEN, therefore suppressing tumor growth and augmenting immunotherapy efficacy (Ren et al., 2019). Recently, Zhang et al. revealed that EVs loaded with miR-130b-3p mediate communication between GC cells and M2 macrophages in the tumor microenvironment by modulating MLL3 and GRHL2 (Zhang Y. et al., 2020). According to data from the TCGA database, lncRNA RP11-323N12.5 is identified as the most significantly upregulated lncRNA in GC. RP11-323N12.5 has been shown to enhance YAP1 transcription in GC cells and further contribute to immunosuppression by promoting MDSC infiltration and Treg cell differentiation (Wang J. et al., 2021). LncRNA microarray detection identified the overexpression of linc-POU3F3 in Treg cells between GC patients and controls. Linc-POU3F3 promotes the distribution of Tregs via activating the TGF-β signaling pathway, leading to GC cell proliferation (Xiong et al., 2015). These findings emphasize that ncRNAs are promising candidates in developing immune anticancer therapeutics.

### Stemness of Gastric Cancer

Cancer stem cells (CSCs) refer to a subset of tumor cells that have the capacity to self-renew and generate diverse cells that constitute the whole tumor (Reya et al., 2001). Emerging evidence has suggested that CSCs play a crucial role in tumorigenesis, metastasis, drug resistance, and tumor relapse (Visvader and Lindeman, 2008). In recent years, ncRNAs were identified to participate in the formation and maintenance of CSCs in cancers, including GC. A miRNA microarray analysis showed that miR-196a-5p is highly expressed in CD44^+^ cells, and its inhibition could reduce colony formation and invasion of GC stem cells (GCSCs) via targeting Smad4 (Pan et al., 2017). By contrast, the expression of miR-98 is decreased in CD44^+^ GCSCs. Elevated miR-98 expression inhibits the ability of self-renewal, tumorigenicity, and invasion of GCSCs by targeting BCAT1 (Zhan et al., 2021). MiR-375 acts as a tumor-suppressive miRNA that reduces GC cell stemness primarily via triggering SLC7A11-dependent ferroptosis (Ni et al., 2021). Increased expression of miR-501-5p is observed in GC tissues and cell lines. MiR-501-5p induces the activation of the Wnt/β-catenin signaling pathway by directly targeting NKD1, GSK3β, and DKK1, which enhances the stemness phenotype in GC (Fan et al., 2016). Azimi et al. evaluated the expression pattern of miRNAs in gastrospheres and GC tissues. High levels of miR-200c-3p and miR-520c-3p were found to affect the stemness and metastasis of GC cells (Azimi et al., 2021). FEZF1-AS1 is significantly increased in GC tissues and associated with sphere formation and expression of stemness markers. FEZF1-AS1 knockdown attenuates the proliferation, migration, and viability of GCSCs via the miR-363-3p/HMGA2 axis (Hui et al., 2020). On the basis of GEO data and qRT-PCR analysis, lncRNA ASB16-AS1 is recognized as an oncogene in GC. High levels of ASB16-AS1 strengthen the stem cell-like characteristics of GC cells. Mechanistically, ASB16-AS1 activates the NF-κB pathway via inducing TRIM37 phosphorylation to facilitate tumor growth, stemness, and cisplatin (CDDP) resistance in GC (Fu et al., 2021). As a tumor suppressor, lncRNA ADAMTS9-AS2 inhibits the tumorigenicity of GCSCs by modulating SPOP expression (Wang F. et al., 2020). Recently, Song et al. discovered that THOR contributes to the stemness of GC cells by increasing stemness marker SOX9 mRNA stability (Song et al., 2018). Sun et al. also suggested that high expression of lncRNA SNHG3 is correlated with the acquisition of stem cell-like properties of GC cells. SNHG3, induced by IL-6/STAT3 transactivation, predominantly plays its oncogenic properties in GC by modulating the miR-3619-5p/ARL2 axis (Sun B. et al., 2021). The role of circRNAs in GC stemness has not been fully deciphered, but preliminary mechanistic studies are beginning to emerge. Upregulated circFAM73A expression is identified in GC tissues and predicts poor survival in GC patients. High levels of circFAM73A increase CSC-like properties via regulating the miR-490-3p/HMGA2 pathway, therefore leading to GC malignancy (Xia et al., 2021). In EBV-associated GC, circLMP2A, which is derived from exon 3 to exon 5 of the LMP2A gene, is remarkably overexpressed in CSCs. CircLMP2A plays an essential role in inducing and maintaining stem cell-like phenotype by targeting the miR-3908/TRIM59/p53 axis in EBV-associated GC cells (Gong et al., 2020). Furthermore, Zhao et al. discovered a novel circRNA (circNOTCH1), which is well expressed in GC tissues and cells. Functional studies demonstrated that circNOTCH1 promotes metastasis and stemness via the miR-449c-5p/MYC/NOTCH1 axis (Zhao et al., 2020).

### Drug Resistance

For advanced GC patients, chemotherapy is the first-line therapeutic strategy to inhibit the progression of GC (Zhang X. et al., 2020). However, chemoresistance is a primary challenge in clinics and scientific research, leading to the recurrence and metastasis of tumors. Although the underlying mechanism of chemosensitivity and chemoresistance is complex, ncRNAs have received increased appreciation to overcome this obstacle (Wang W.-T. et al., 2019). CDDP is one of the most widely used chemotherapeutic drugs in the treatment of various tumors, including GC (Chen and Chang, 2019; Smyth et al., 2020). Nevertheless, after several cycles of CDDP-based treatment, patients always exhibit acquired drug resistance. Accumulating evidence revealed that various ncRNAs play crucial roles in mediating CDDP resistance in GC (Poursheikhani et al., 2020). For example, miR-21 is overexpressed in the CDDP-resistant cell line SGC7901/DDP. Mechanistically, miR-21 induces CDDP resistance via inhibiting the expression of PTEN and activation of the Akt pathway (Yang et al., 2013). A microarray profiling study identified miR-99a and miR-491, which are highly expressed in CDDP-resistant GC cell lines. Oncogenic miR-99a and miR-491 enhance CDDP resistance of GC cells by directly suppressing their target gene CAPNS1 (Zhang et al., 2016). Similarly, the overexpression of miR-223 promotes CDDP resistance of GC cells by modulating the cell cycle via targeting FBXW7 (Zhou et al., 2015). By contrast, miR-129 is downregulated in CDDP-resistant GC samples and cells. MiR-129 knockdown reduces chemosensitivity to CDDP via suppressing the expression of P-gp in GC cells (Lu et al., 2017). As a tumor suppressor, miR-7 also increases CDDP sensitivity via targeting mTOR in GC cells (Xu N. et al., 2017). Furthermore, the expression level of lncRNA HOTAIR is higher in CDDP-resistant GC cells than in parental GC cells. HOTAIR can contribute to CDDP resistance of GC cells via sponging endogenous miR-34a to inhibit PI3K/Akt and Wnt/β-catenin signaling pathways (Cheng et al., 2018). Ye et al. found that HOXD-AS1 leads to CDDP resistance in GC epigenetically by silencing PDCD4 expression (Ye et al., 2019). FOXD1-AS1 is also highly expressed in GC cell lines, and its overexpression increases the resistance of GC cells to CDDP. Mechanistic evidence revealed that FOXD1-AS1 promotes the translation of FOXD1 via PIK3CA/PI3K/AKT/mTOR signaling, therefore inducing GC chemotherapy resistance (Wu et al., 2021). Wang et al. identified a novel lncRNA, cisplatin resistance-associated lncRNA (CRAL), which acts as a ceRNA to reverse CDDP resistance in GC via the miR-505/CYLD/AKT axis (Wang Z. et al., 2020). ADAMTS9-AS2 functions as a tumor suppressive lncRNA and sensitizes GC cells to CDDP by inducing NLRP3-mediated pyroptotic cell death via downregulating miR-223-3p (Ren et al., 2020). To study the role of circRNAs in CDDP resistance, Sun et al. profiled the differentially expressed circRNAs between CDDP-sensitive and CDDP-resistant GC cells using RNA sequencing. The circRNA MCTP2 is downregulated in CDDP-resistant GC tissues and cells. High levels of circMCTP2 sensitize GC cells to CDDP through miR-99a-5p-mediated induction of MTMR3 expression (Sun et al., 2020). CircAKT3 (hsa_circ_0000199), identified by RNA sequencing, also plays a crucial role in the DDP resistance of GC. Mechanistic evidence showed that circAKT3 knockdown could effectively increase CDDP sensitivity in GC cells via targeting the miR-198/PIK3R1 axis (Huang et al., 2019). CircDONSON is also well expressed in GC tissues and cells. The silencing of circDONSON reduces CDDP resistance of GC cells by modulating the miR-802/BMI1 axis (Liu et al., 2020). Moreover, circCUL2 functions as a regulator of CDDP sensitivity via miR-142-3p/ROCK2-mediated autophagy activation (Peng L. et al., 2020).

Furthermore, the development of multidrug resistance (MDR) of tumor cells is a primary impediment in clinical oncology, leading to poor prognosis in GC patients (Wei L. et al., 2020). Recently, ncRNAs have received attention as essential regulators in the MDR of GC through multiple mechanisms. For example, An et al. discovered a novel subset of MDR-associated miRNAs through high-throughput sequencing. Among them, miR-23b-3p was further demonstrated to play essential roles in the MDR of GC. The overexpression of miR-23b-3p suppresses autophagy in MDR cells by targeting HMGB2 and ATG12 and enhances chemotherapy sensitivity of MDR cells (An et al., 2015). Chen et al. demonstrated that miR-495-3p is downregulated in MDR cell lines. High levels of miR-495-3p also inhibit the MDR of GC cells by targeting GRP78 and modulating the autophagy process (Chen S. et al., 2018). In a high-throughput sequencing study, miR-508-5p was identified as an MDR-related miRNA. *In vitro* and *in vivo* experiments revealed that miR-508-5p can reverse GC cell resistance to multiple chemotherapeutics by suppressing the expression of ABCB1 and ZNRD1 (Shang et al., 2014). Similarly, miR-19a/b is overexpressed in MDR GC cell lines. Mechanistic evidence suggested that miR-19a/b regulate the MDR of GC cells via the PTEN/AKT signaling pathway (Wang et al., 2013). Additionally, the overexpression of miR-106a induces MDR via the downregulation of RUNX3, and miR-1 reverses MDR in GC by inhibiting sorcin expression (Zhang et al., 2013; Deng et al., 2019). To explore the function of lncRNAs in MDR, Wang et al. analyzed the differentially expressed lncRNAs between the MDR sublines and SGC7901 using RNA microarrays. LncRNA NR_024,549 (termed MRUL) is remarkably upregulated in MDR GC cell sublines. MRUL plays an enhancer-like role in upregulating P-gp expression, leading to the development of MDR (Wang et al., 2014). High expression of lncRNA GHET1 and PVT1 in GC cells also contribute to the development of MDR (Zhang et al., 2015; Zhang X. et al., 2017). Furthermore, Liu et al. found a novel oncogenic lncRNA FENDRR associated with MDR in GC. Mechanistically, the downregulation of FENDRR decreases drug resistance of MDR GC cells by sponging miR-4700-3p to promote FOXC2 expression (Liu H. et al., 2021).

## NcRNAs as Biomarkers in Gastric Cancer

MiRNAs are generally stable in the serum, plasma, and other body fluids because of their ability to escape from endogenous ribonuclease-mediated degradation and their small size (Witwer, 2015; Shigeyasu et al., 2017). Multiple studies have underscored the diagnostic and prognostic efficiency of serum or plasma-based miRNA levels (Shigeyasu et al., 2017). Therefore, circulating miRNAs have the potential to be promising noninvasive biomarkers in patients with GC for early detection and surveillance of disease progression. In an miRNA profiling study, Zhu et al. screened five upregulated miRNAs in serum from GC patients. The combination of miR-92a, miR-486-5p, miR-16, miR-25, and miR-451 could differentiate early-stage GC patients from healthy controls with an area under the curve (AUC) of 0.890 (Zhu et al., 2014). In another study, Yan So et al. developed and validated a serum 12-miRNA biomarker panel assay, which might be a valid risk assessment tool for detecting GC (discovery cohort: AUC = 0.93, and validation cohort: AUC = 0.92) (So et al., 2021). Recently, Abe et al. revealed that a novel combination of serum miRNAs (miR-187-5p, miR-4257, miR-5739, and miR-6785-5p) could achieve a high diagnostic power in separating early-stage GC cases from non-cancer controls (discovery set: AUC = 0.996, and validation set: AUC = 0.998) (Abe et al., 2021). Circulating miRNAs also have prognostic value. For example, serum miR-203 expression is remarkably reduced in GC patients with vessel invasion, a higher T, lymph node metastasis, and distant metastasis. Low expression of serum miR-203 is positively correlated with poor disease-free and overall survival, implying that miR-203 is a noninvasive biomarker for predicting the prognosis and metastasis in GC patients (Imaoka et al., 2016). Tsai et al. discovered that elevated circulating miR-196a/b levels are significantly associated with more advanced stages and poorer survival of GC. Additionally, the levels of circulating miR-196a/b are decreased after surgical resection in GC patients, suggesting the potential value of miRNA biomarkers for tumor monitoring (Tsai et al., 2016). Taken together, these studies highlight the significant potential of circulating miRNAs in the diagnosis and prognosis of GC.

LncRNAs can also be released into the extracellular environment and can subsequently be detected in serum, plasma, and other bodily fluids as circulating lncRNAs (Anfossi et al., 2018). Circulating lncRNAs and their potential applications as biomarkers for cancer diagnosis and prognosis are currently subjects of increasing research interest (Qi et al., 2016). For example, the efficacy of serum lncRNA CTC-497E21.4 was analyzed for GC diagnosis and was found to be able to differentiate GC patients from healthy controls (AUC = 0.848). The expression levels of lncRNA CTC-497E21.4 are significantly reduced after surgical resection, implying its potential application in tumor dynamic monitoring and prognosis prediction (Zong et al., 2019). Three serum lncRNAs (PTENP1, LSINCT-5, and CUDR) were identified to be promising diagnostic markers for GC. The risk model for these three lncRNAs demonstrated that healthy individuals could be distinguished from early GC patients (Dong et al., 2015). The expression of lncRNA SNHG5 in the serum is remarkably reduced in GC patients. The dysregulation of lncRNA SNHG5 can be a promising biomarker (AUC = 0.904) for GC diagnosis (Li et al., 2021). In addition to exploring the diagnostic and prognostic values of well-characterized lncRNAs in GC, a few studies have been carried out to identify novel circulating lncRNA biomarkers. In a genome-wide lncRNA screening analysis, Zhang et al. discovered five novel plasma lncRNAs, including LINC00857, AOC4P, BANCR, CCAT2, and TINCR. The combination of these five lncRNAs achieves an AUC of 0.91 for discriminating GC patients from healthy controls. Furthermore, this five-lncRNA signature could distinguish GC patients from patients with gastrointestinal stromal tumor and precancerous individuals with AUCs of 0.80 and 0.82, respectively (Zhang K. et al., 2017). In another study, Liu et al. identified that three novel lncRNAs (CTC-501O10.1, AC100830.4, and RP11-210K20.5) are upregulated in the plasma of GC patients. A panel of these three circulating lncRNAs could be used to distinguish between healthy individuals and patients with GC (AUC 0.764) (Liu J. et al., 2018). The diagnostic and prognostic values of circulating circRNAs in GC are not completely deciphered, but relative studies are emerging: circ0001821 is significantly downregulated in whole-blood specimens from GC patients. The dysregulation of circulating circ0001821 was identified as a promising biomarker (AUC = 0.872) for GC diagnosis (Kong et al., 2019). Circ_0002874 expression is remarkably increased in the plasma of GC patients, and related to the tumor stage and lymph node metastasis. This circRNA achieves a high diagnostic power in separating GC patiets from non-cancer controls (AUC = 0.836) (Sun X. et al., 2021). Circulating ncRNAs with potential diagnostic or prognostic role in GC are listed in Table 2.

**TABLE 2:** Circulating ncRNAs with potential diagnostic or prognostic role in GC.

RNA species	Name	Expression	Source	Biomarker	References
miRNA	miR-92a, miR-486-5p, miR-16, miR-25, and miR-451	Up	Serum	Diagnosis	Zhu et al. (2014)
	twelve miRNA panel	Unknow	Serum	Diagnosis	So et al. (2021)
	miR-187-5p, miR-4257, miR-5739, and miR-6785-5p	Unknow	Serum	Diagnosis	Abe et al. (2021)
	miR-203	Down	Serum	Prognosis	Imaoka et al. (2016)
	miR-196a/b	Up		Prognosis	Tsai et al. (2016)
lncRNA	CTC-497E21.4	Up	Serum	Diagnosis and prognosis	Zong et al. (2019)
	PTENP1, LSINCT-5, and CUDR	Down	Serum	Diagnosis	Dong et al. (2015)
	lncRNA SNHG5	Down	Serum	Diagnosis	Li et al. (2021)
	LINC00857, AOC4P, BANCR, CCAT2, and TINCR	Up	Plasma	Diagnosis	Zhang et al. (2017a)
	CTC-501O10.1, AC100830.4 and RP11-210K20.5	Up	Plasma	Diagnosis	Liu et al. (2018a)
circRNA	circ_0001821	Down	Whole blood	Diagnosis	Kong et al. (2019)
	circ_0002874	Up	Plasma	Diagnosis	Sun et al. (2021b)

Furthermore, according to the ClinicaTrials.gov database, several ncRNA diagnostic clinical trials are also underway in GC. For example, one clinical trial uses various blood biomarkers, including miRNAs, to screen with a high risk of GC individuals (NCT04329299). In order to predict the chemotherapy response in GC, patients are recruited for developing a miRNA and mRNA model in the other clinical trial (NCT03253107). Furthermore, another clinical trial evaluates the expression of lncRNATHRIL and lncRNA PACER in patients with gastrointestinal disease (such as GC) based on H. pylori infection (NCT03057171). The information of clinical trials is listed in Table 3. The discovery of numerous ncRNAs, their widespread expression patterns, their stability in circulating body fluids, and their specificity provide promising diagnostic and prognostic biomarkers in cancer. However, the development of ncRNAs as liquid biomarkers in GC is challenged by several limitations. One is the specificity and sensitivity of ncRNAs. The huge numbers of lncRNAs are differentially expressed in various types of cancer, and their expression also varies between different human populations (Telonis and Rigoutsos, 2018; Slack and Chinnaiyan, 2019). It is important to determine the most crucial ncRNAs in a specific subtype of GC, and further to validate GC-specific ncRNA expression in different ethnicities. In addition, ncRNAs can be detected in various bodily fluids of GC, including blood, serum, plasma, feces, gastric juice, or saliva (Weber et al., 2010). The development of optimal isolation procedures, efficient detection technologies, and specific delivery methods are also critical to facilitate the specificity and sensitivity of ncRNAs in the GC diagnosis. Another limitation is the structure and function of ncRNAs. The field of ncRNAs is still in its infancy, and the functional characterization of most ncRNAs remains unknown. It is necessary to identify the role and molecular mechanism of ncRNAs in GC, and to construct the cause–effect relationship of each ncRNA for determining their specificity.

**TABLE 3:** Clinical trials of ncRNAs in GC.

ClinicalTrials.gov identifier	Official title	Biomarker	Source	Population
NCT04329299	Use of Blood Biomarkers to Predict Gastric Cancer Risk	miRNA	Blood	6,862 participants
NCT03253107	Predicting Biomarker of Gastric Cancer Chemotherapy Response	miRNA	Blood and tissue	800 participants
NCT03057171	A Study on the Gastrointestinal Disease and Helicobacter Pylori Controlled Long Non-coding RNA	LncRNA	Tissue	50 participants

## NcRNA as Therapeutic Agents or Targets

The ability of ncRNAs to modulate gene expression makes them potential efficacious targets for tumor therapy (Toden et al., 2021). Generally, there are two main approaches for targeting ncRNAs in cancer therapy: restoration of tumor-suppressive ncRNAs and inhibition of oncogenic ncRNAs (Xie S. et al., 2020). NcRNA inhibition therapy can be achieved by using antisense oligonucleotides (ASOs), small-molecule inhibitors, and miRNA sponges (artificial circRNAs) (Shah et al., 2016; Arun et al., 2018; Dragomir et al., 2020). ASOs are short, synthetic, single-stranded oligonucleotides with the ability to complementary bind and inhibit RNA molecules (Shah et al., 2016). In a recent study, Li et al. reported that lncRNA PVT1 is highly expressed in gastric adenocarcinoma tissues and positively associated with larger tumor size, deeper invasion depth, and poor survival. *In vitro* and *in vivo* PDX models have shown that PVT1 ASOs significantly inhibit PVT1 expression and exhibit obvious antitumor activity (Li Y. et al., 2020). ASOs targeting miRNA are termed anti-miRNA oligonucleotides (AMOs). Lima et al. used the model of GC as a proof-of-concept to design locked nucleic acid-based AMOs capable of silencing oncogenic miR-9 expression (Lima et al., 2018). Small-molecule inhibitors are also designed to suppress the function and biogenesis of oncogenic ncRNAs. Vo et al. developed small-molecule inhibitors of oncogenic miRNA (miR-373 and miR-372) in GC via chemically modifying neomycin. They are able to specifically bind pre-miR-373 and pre-miR-372 and subsequently inhibit the processing of these oncogenic miRNAs by Dicer enzyme (Vo et al., 2014). Furthermore, miRNA sponges are synthetic RNA molecules that contain multiple artificial miRNA binding sites to bind and sequester miRNAs (Ebert et al., 2007). CircRNAs can sponge several miRNAs simultaneously and have higher stability than other RNA species, making them an ideal miRNA sponge (Dragomir and Calin, 2018). MiRNA sponges in GC are not yet developed, but new drugs can be created. In addition, ncRNA mimetic molecules are a valid alternative to restore the tumor suppressive function of ncRNAs via replacing the lost ncRNA using synthetic molecules (Shah et al., 2016; Xie S. et al., 2020; Dragomir et al., 2020).

## Conclusion

NcRNAs are emerging as crucial players in cell biology and regulation of gene expression. Although the contribution of ncRNAs to the genesis and progression of cancer has attracted growing interest, more efforts are needed to explore the full extent of their contribution and the detailed functional mechanisms. Recently, advances in RNA-sequencing techniques and bioinformatic tools have promoted the identification and expression profiling of numerous ncRNAs in GC cells. Many of them have been reported to play an essential role in GC-related signaling pathways. In this review, we summarize ncRNAs that have been experimentally validated to affect the hallmarks of GC by regulating critical pathways. A better understanding of ncRNA regulation will contribute to reveal the molecular mechanism of gastric carcinogenesis and progression. Furthermore, the promising applications of ncRNAs as diagnostic biomarkers and therapeutic targets for GC patients have been exemplified by the studies highlighted in this review.

Accumulating evidence has suggested that various ncRNAs are involved in different cancer-related processes, especially in tumor immune escape, drug resistance, and stemness. We expect that further studies will advance our knowledge of ncRNAs in GC and clarify the role of ncRNA in cancer heterogeneity and intratumoral clonal evaluation. Furthermore, more investigations are required to unravel the sophisticated crosstalk of ncRNAs between cancer-associated fibroblasts, immune cells, endothelial cells, and tumor cells in the tumor microenvironment. Methodologies that can be utilized to determine temporal and spatial gene expression patterns in specific cell types or subclones, particularly at the single-cell level, will contribute to this endeavor. Single-cell RNA sequencing may provide a compelling approach to deciphering the biology of ncRNAs in GC. In the past few years, ncRNAs have been proposed as potential diagnostic biomarkers and therapeutic targets in GC. Circulating ncRNAs and their potential applications as noninvasive biomarkers for GC detection and molecular classification are currently subjects of increasing research interest. Further research is required to validate the sensitivity and specificity of ncRNAs in prospective studies with standardized methodology as well as extensive and diverse populations. The application of ncRNAs as targets also holds great promise for the treatment of GC. Identification of optimal ncRNA targets is an essential component for therapeutic development; thus exploration of the molecular biology in GC-specific content is necessary. Currently, a few miRNA targeted therapeutics have been tested in clinical trials. New ncRNA-targeting drugs with high efficacy and specificity will be available for clinical use in the future. One of the biggest challenges of ncRNA targeted therapeutics is how to take the agents specifically to GC cells without inducing adverse reactions in other cells. With the development of nanotechnology and chemical technology, the ingenious carriers (such as nanoparticles) which are specifically designed for delivery to GC cells, will contribute to solve the problem. Nevertheless, further translation studies and clinical trials are needed to promote the development of ncRNA-based diagnostic biomarkers and therapeutic targets to benefit GC patients.
